# Interactions of Equine Viruses with the Host Kinase Machinery and Implications for One Health and Human Disease

**DOI:** 10.3390/v15051163

**Published:** 2023-05-13

**Authors:** Carol Anderson, Haseebullah Baha, Niloufar Boghdeh, Michael Barrera, Farhang Alem, Aarthi Narayanan

**Affiliations:** 1 School of Systems Biology, College of Science, George Mason University, Fairfax, VA 22030, USA; cander47@gmu.edu (C.A.); hbaha@gmu.edu (H.B.); mbarrer@gmu.edu (M.B.); 2 Institute of Biohealth Innovation, George Mason University, Fairfax, VA 22030, USA; nboghdeh@gmu.edu (N.B.); falem@gmu.edu (F.A.); 3 Department of Biology, College of Science, George Mason University, Fairfax, VA 22030, USA

**Keywords:** equine viruses, zoonotic pathogens, intermediate host, mosquito vectors, encephalitic disease, kinases, inhibitors

## Abstract

Zoonotic pathogens that are vector-transmitted have and continue to contribute to several emerging infections globally. In recent years, spillover events of such zoonotic pathogens have increased in frequency as a result of direct contact with livestock, wildlife, and urbanization, forcing animals from their natural habitats. Equines serve as reservoir hosts for vector-transmitted zoonotic viruses that are also capable of infecting humans and causing disease. From a One Health perspective, equine viruses, therefore, pose major concerns for periodic outbreaks globally. Several equine viruses have spread out of their indigenous regions, such as West Nile virus (WNV) and equine encephalitis viruses (EEVs), making them of paramount concern to public health. Viruses have evolved many mechanisms to support the establishment of productive infection and to avoid host defense mechanisms, including promoting or decreasing inflammatory responses and regulating host machinery for protein synthesis. Viral interactions with the host enzymatic machinery, specifically kinases, can support the viral infectious process and downplay innate immune mechanisms, cumulatively leading to a more severe course of the disease. In this review, we will focus on how select equine viruses interact with host kinases to support viral multiplication.

## 1. Introduction

Zoonotic diseases, which constitute a large number of (re)emerging viral infections globally, are transmitted to humans from their natural animal reservoirs by intermediate vector species, including other animals such as bats and swine, and insects such as mosquitoes and sandflies [1]. It is currently estimated that more than half of all human pathogens originate from animal hosts with approximately 78% of emerging human pathogens being zoonotic [1,2]. Spillover events from animals to humans appear to be increasing as humans interact more directly with livestock, as well as wild and domesticated animals [3]. Additionally, urbanization and deforestation have forced indigenous species from existing habitats and into closer contact with humans [1]. It is also becoming prominently clear that climate change has a major impact on (re)emerging zoonotic viral pathogens [4]. The emergence of HIV in the 1920s from human interaction with infected bush meat derived from wild primates is possibly one of the best examples of how a zoonotic virus can establish itself in the human population and evolve into a global challenge [3]. It is estimated that more than 60 million people have contracted HIV since its emergence, and the virus has been linked to 25 million deaths [5]. While therapeutic solutions are available to treat HIV infection, the global epidemiological data indicate that there is a continual rise in infected individuals, further exacerbated by the failure to deliver an effective vaccine solution for HIV [6,7].

Equine viruses represent a major concern for human disease because of the potential for equines to serve as intermediate hosts [1]. The viremic load achieved by several equine viruses is extremely high, and thus the potential for spillover mediated by insect vectors is significantly increased [8]. Examples of such equine viruses that have rampantly spread in the human population, transmitted by insect vectors, include West Nile virus (WNV) and the equine encephalitis viruses (EEVs) that have continued to spread to new areas of the globe in recent years [1,9,10,11]. In addition to natural vector-based transmission, other economic factors, such as increased travel and trade of equines between countries, can introduce new diseases in non-native areas [1]. Notably, even for viruses that were not traditionally associated with specific hosts, emerging data seem to suggest new reservoirs. For example, studies suggest that viruses such as the Middle Eastern respiratory syndrome coronavirus (MERS-CoV) could potentially infect horses, leaving open a new possible reservoir [12]. From a One Health perspective, there is an undeniable need for understanding the spillover risks associated with zoonotic pathogens, and how they affect their animal reservoirs, eventually contributing to human disease and the global disease burden. 

A step towards further understanding how equine viruses play a role in human disease is to elucidate how the host machinery is engaged by these viruses, both in the equine and the human hosts. Host kinases constitute the cellular enzymatic machinery that aids in the transferal of phosphate groups from ATP to other molecules that results in the functionalization of the phosphorylated target. Such functionalized targets directly result in phosphosignaling cascades that control key cellular events such as growth, differentiation, and maturation. Kinases phosphorylate a subset of host targets: tyrosine, serine/threonine residues on proteins, or lipids [13,14]. Several such phosphorylated and functionalized kinases play critical roles in innate immune mechanisms that sense invading viruses and mount an antiviral response. RNA-dependent protein kinase R (PKR) is one such kinase that is vital for the host detection of double-stranded non-coding RNA [13]. PKR will bind to the N-terminal of the RNA, which then undergoes phosphorylation that results in the prevention of the translation of the viral RNA. 

Viruses have in turn developed mechanisms that can evade, moderate, and/or nullify the host-based innate immune mechanisms by controlling the host processes such as transcription and translation. Such virus-based control of host innate immune mechanisms often involves interactions between viral and host proteins. There are a wide variety of zoonotic viruses that have evolved escape mechanisms from PKR to enable and sustain robust infection [13]. One such example is the Rift Valley fever virus (RVFV), a zoonotic pathogen of high relevance to One Health, in which case the non-structural protein NSs are known to bind to PKR and cause the degradation of PKR [15,16]. Viruses are obligate pathogens and hence depend on the host machinery to establish a productive infection, in addition to evading the innate immune response. A variety of kinases have been demonstrated to be directly essential for the infectious process, including viral entry, nucleic acid synthesis, and packaging, including protein kinase C (PKC), extracellular signal-regulated kinase (ERK), phosphatidylinositol-3-kinase (PI3K), and focal adhesion kinase (FAK). Other kinases such as Ikappa B kinase (IKK), c-Jun-N-terminal kinase (JNK), and p38 mitogen-activated protein kinase (MAPK) have been shown to be involved in the upregulation of inflammatory mediators (chemokines and cytokines) in response to infection [13]. The understanding of host kinases and their interaction with viruses opens the door to the development of host-targeted inhibitors against these viruses, which inherently brings the advantages of being broad-spectrum and of low potential for the emergence of viral resistance [17,18]. Through this review, we will highlight some important kinases that are involved in interactions with equine viruses, and how these interactions influence the infection process. 

## 2. Equine Encephalitis Virus (EEV)

In this section, we will describe the interactions of two closely related EEVs with host-derived kinases, specifically focusing on the Venezuelan equine encephalitis virus (VEEV) and eastern equine encephalitis virus (EEEV). VEEV and EEEV are new-world alphaviruses belonging to the family *Togaviridae*. These single-stranded RNA viruses are the causative agents for encephalitic disease in equines and humans in the Americas [9,10,11,19]. While human mortality associated with this mosquito-transmitted virus is relatively low for VEEV, morbidity and mortality are significantly higher (45–75%) in the case of EEEV. In both cases, the acute disease is characterized by encephalitis, with the potential for long-term neurological sequelae in survivors [19]. VEEV is characterized by a high viremic load that can be transmitted to other hosts via mosquito bites. EEEV does not result in a high viremic load, the result of which is that humans and equines are considered dead-end hosts, where the produced virus is unable to adequately infect mosquitoes and continue viral circulation [20]. Birds are the primary hosts for VEEV and EEEV, while horses and donkeys serve as intermediate hosts and act as reservoirs in the environment. Unvaccinated horses are highly susceptible to infection and associated equine disease, often serving the role of sentinels for epidemiological monitoring. In the context of infected equines, symptoms of the disease include fever, behavioral changes such as depression, head pressing or circling, muscle twitches, paralysis, and eventual death of the animal [21]. The treatment of infected horses at this stage is only supportive, with a high ensuing mortality rate. An equine EEV vaccine is available as a cocktail of encephalitic viral antigens and is highly effective in preventing equine disease and death. 

VEEV and EEEV are major concerns for human disease not only because of the potential for transmission to humans through infected mosquitoes but also because these viruses are highly stable and infectious in aerosols form. In the context of aerosol transmission, there is a significant involvement of the central nervous system (CNS) and a high potential for encephalitic disease and death. In the context of human disease, several studies have been conducted about the interaction of VEEV proteins with human kinases and how they contribute to the establishment of a productive infection. For example, the human IKKβ kinase was shown to play an important role in the ability of VEEV to multiply in the cells of CNS origin through a series of studies involving small-molecule inhibitors and targeted depletion using siRNAs, as well as through additional proteomic studies [22,23,24]. The requirement of IKKβ kinase for EEV multiplication in infected cells is suggested to be broad-spectrum as the inhibitors of this kinase elicit the robust inhibition of all new world encephalitic alphaviruses. Of significance are the published studies that suggest VEEV non-structural protein (nsP3) could be a target for the IKKβ-mediated phosphorylation and mutation of the phosphorylated residues in nsP3, resulting in the abrogation of the infection [22,23,24]. IKKβ is an integral component of the nuclear factor kappa B (NF-κB) signaling cascade. Other inhibitors that target members of this signal transduction pathway, such as the FDA-approved drug celecoxib, were also observed to inhibit VEEV load in vitro, suggesting that this pathway is an important aspect of the viral multiplication process [25]. Among the cellular stress-related kinases, the PERK enzymatic pathway was shown to be important for VEEV, as the inhibition of the pathway by small molecules and siRNAs resulted in a significant level of downregulation of the viral load, potentially by impacting the translation of non-structural proteins [26,27,28]. Previous studies also demonstrated that the upregulation of early growth response 1 (EGR1) protein in the context of VEEV infection was related to PERK activity [27]. Protein kinase C (PKC) was also shown to interact with the viral capsid protein, which then modulates the phosphorylation of PKC in a manner directly relevant to viral multiplication [26]. Studies using small-molecule inhibitors have demonstrated that among the signal transduction pathways that are related to cell growth and survival, the MEK/ERK MAPK pathway and the AKT pathway are highly relevant to VEEV and EEEV infection in human cells [29,30].

## 3. Equine Arteritis Virus (EAV)

Equine arteritis virus (EAV) is an enveloped, single-stranded, positive-sense RNA virus belonging to the *Arteriviridae* family [31]. EAV is highly specific, infecting almost exclusively equine species, such as horses and zebras. It primarily targets macrophages and small blood vessel endothelial cells for infection. This has been known to lead to decreased macrophage presence in the blood and weaker immune responses. The virus was first isolated in 1953, and was characterized by highly specific symptoms in horses. This includes the development of lesions in the liver and lymph nodes, anorexia, depression, muscle stiffness, conjunctivitis, and respiratory inflammation. This virus has also been known to cause spontaneous abortions in mares, with rates varying between 10% and 70% depending on the viral strain [32]. A small number of stallions can develop chronic infections in their reproductive tract where shedding persists via semen, but there is no evidence that EAV causes chronic infection in mares or foals [31]. The virus can also be transmitted through respiratory droplets when there is close contact, and through the venereal route [32].

While not fully elucidated mechanistically, viral entry is likely to be clathrin-dependent [31,33]. Chronic infections have been connected with the upregulation of interleukin 2 inducible T-cell kinase (ITK), lymphocyte protein tyrosine kinase (LCK), and zeta-chain T-cell-receptor-associated protein kinase 70 (ZAP70). The phosphorylation of protein kinase B showed an interesting difference in abundance between short-term infections and chronic infections [34]. Persistent infection with EAV was also correlated with the TYRO protein tyrosine kinase binding protein (TYROBP) gene and ITK upregulation [34]. NF-κB signaling has been implicated in the EAV infectious process by its impact on the innate immune myeloid differentiation primary response gene 88 (MyD88). The activation of the NF-κB cascade by MyD88 resulted in the expression of type I interferons (IFN I) and cytokines that promote inflammation, thus attesting to the role of this kinase in the evolutionarily conserved interferon-driven antiviral response [35,36]. Casein kinase II (CKII) has also been shown to be involved in the phosphorylation of EAV structural proteins, which affects cellular polarity, viral-induced stress response, and transcription/translation [36]. 

## 4. West Nile Virus (WNV)

West Nile virus (WNV) belonging to the *Flaviviridae* family, is an enveloped, single-positive stranded RNA virus that is transmitted to humans through infected mosquitoes [37]. First isolated in Uganda in 1937, WNV has since spread to the main areas of the globe, first appearing in the United States in 1999 [38]. WNV possibly remains the poster child of how a virus, when introduced in a non-native environment, can establish itself as an endemic pathogen that causes annual disease in animals and humans. Birds and mosquitoes represent the primary reservoirs for WNV. Humans and equines are considered dead-end hosts, as not enough infectious viral particles are produced to infect mosquitoes. WNV is responsible for a majority of encephalitic infections caused by flaviviruses in equines, though Japanese encephalitis virus (JEV) and Murray Valley encephalitis virus (MVEV) have been implicated as well [37]. There are currently four licensed vaccines available for horses, although they have not been approved for human use [39]. WNV remains an area of global concern due to potential economic losses and its impact on public health [39]. WNV has been implicated in neuronal cell death, and an estimated 8% of horses will develop neurological disease following infection, thus leading to encephalitis. This ranges from muscle weakness, depression, and anorexia to lameness, muscle tremors, and blindness [37]. In addition to CNS infection, a variety of organs such as the liver, kidney, spleen, and heart can also be infected by WNV [39]. Estimates of chronic disease manifestations are as high as 20%, including weight loss, lethargy, and nervous system defects [37]. 

Among the kinases that are implicated in WNV infection, AMP-activated protein kinase (AMPK) degradation mediated by the WNV C protein was through ubiquitin-mediated protein degradation. AMPK is a stress-activated protein that inhibits key responses, including autophagy. The ubiquitin-mediated degradation of AMPK is thought to be relevant to the development of the CNS phenotype in WNV infection [40]. Calcium (Ca2+) influx and its associated stress during early viral entry have been linked to the activation of a variety of kinases, including FAK, ERK1/2, and protein-serine kinase B alpha (Akt) [41]. The downregulation of Ca2+ has been linked to lower viral titers, indicating a potential reliance on these pathways for viral replication [41]. Evidence suggests that the WNV capsid protein is able to block apoptosis by targeting the PI3K pathway. High levels of prosurvival kinase Akt, which is downstream of the PI3K pathway, have been linked to WNV infection as well as other flaviviruses [42]. The inhibition of these mechanisms allows for adequate time for viral replication and decreased CD8+ T-cell response by Fas ligation [42,43]. Innate immune interferon (IFN) responses regulated by the JAK/STAT pathway have been implicated in animal survival in murine WNV challenge studies, although the exact mechanism has not been well described. It is hypothesized that WNV non-structural proteins bind to IFN receptors Jak1 or Tyk2, and inhibit them, or possibly suppress cytokine signaling through the SOCS3 pathway, further implicating ubiquitin-mediated signaling events in WNV pathology [44]. PKCs have been linked with decreased WNV and other flavivirus (such as dengue virus) replication in cell culture models. Calaphostin C and chelerythrine, which are PKC inhibitors, have been shown to be inhibitory to WNV replication while also preserving cell viability in cell culture models [45].

## 5. Equine Infectious Anemia Virus

Equine infectious anemia virus (EIAV) is an enveloped, RNA virus belonging to the *Retroviridae* family. Acute infection with EIAV usually presents with recurrent high fever, depression, anorexia, and anemia [46]. Spontaneous abortions have been known to occur in mares infected during pregnancy. Characteristic of being a retrovirus, EIAV infection results in chronic infections in all cases, though often there are no clinical manifestations [46]. Recurrent fevers coincide with a high viral load during episodes of illness [47]. Although the disease is not frequently fatal, it can be in rare cases. EIAV can be transmitted to equines via interaction with infected blood, such as through transfusions. More importantly, EIAV has been shown to spread through insects such as horseflies or deerflies [48]. Mosquitoes, on the other hand, are unlikely to serve as transmission vectors [48]. New evidence shows the spread of EIAV, including its presence in donkey populations in Brazil [49]. Humans can be infected with EIAV via occupational exposure, such as through needle stick injury, when providing care for infected equines [8]. While EAIV has been used as an animal model for describing lentivirus infections in humans, there is no evidence to suggest that humans can become infected with EIAV through natural routes [50]. 

Limited information exists on the interactions of EIAV with host kinases. The presence of equine lentivirus receptor 1 (ELR1) on equine cells appears to make cells susceptible to EIAV infection [51]. ELR1 has been implicated as the sole receptor for EIAV entry into host cells [52]. ELR1 belongs to the tumor necrosis factor receptor (TNFR) protein family, which contributes to the downstream signaling of NF-κB and MAPKs [53]. Positive transcription elongation factor b (P-TEFb) binds to RNA polymerase II through cyclin T and cyclin-dependent kinases 9 (CDK9) [54]. T1 is the predominant cyclin T and has been shown to interact with the viral Tat protein in HIV-1 infections, allowing for viral RNA transcription [55]. For this to take place, it is necessary to recruit human or equine cyclin T1 (eCT1) as a cofactor. This similar mechanism makes studying EAIV of particular importance for HIV infections in humans, and a successful mouse model exists for transgenic mice with both ELR1 and eCT1 [56].

## 6. Group A Rotavirus (RVA)

Group A rotavirus (RVA) is a double-stranded RNA virus of the family *Reoviridae*. RVA infection is the leading cause of diarrhea-associated morbidity and mortality globally among infants and children under the age of 5. Although RVA-associated deaths are preventable via vaccine, the hospitalization rates of children under the age of 5 due to RVA are reported to be 30–40% worldwide. Rotavirus was first discovered in the 1950s from anal swaps of monkeys, and in the 1960s, it was seen again in the gastrointestinal tracts of mice. In 1973, Ruth Bishop and colleagues had a breakthrough in identifying RVA in samples from children with acute diarrhea. The distinct appearance gave its name rotavirus (adapted from the Latin word “rota” meaning wheel) [57]. The transmission of RVA follows the fecal–oral route. The fecal–human spread is mainly facilitated by environmental reservoirs such as fluids, food, fingers, and fomites through the interactions of humans or animals with their environments. In addition, flies as a natural process can also spread RVA shed in feces. Spreads of the virus are quite easy among children and from infected children; transmission to close contacts is possible. In affected persons, acute illness is usually characterized by the early stage of the disease, which subsequently results in milder illness with no visible symptoms in some individuals. In adults, asymptomatic infections can lead to viral transmission to close contacts. The frequent exposure of susceptible children in daycare centers and family daycare homes usually facilitates RVA transmission [58]. 

While RVA has gained significant attention as a human pathogen due to the devastating impact it has on unvaccinated children, equine RVA is also a major pathogen that causes diarrhea in foals [59]. Mortality is low in foals aged 6 months or younger, where cases typically are seen, but more severe disease and death are common in foals < 2 weeks of age. The mode of transmission is akin to that in humans, primarily by the oral–fecal route. Notably, equine RVA is a non-enveloped virus, making it more resistant to disinfectants, thus also contributing to disease spread, persistence, and severity in foals. The virus can be detected in the fecal samples of infected animals for as long as 12 days, even if the animals do not manifest clinical symptoms. The clinical signs of disease in foals, similar to humans, are diarrhea and associated dehydration, inconsistent fever, lethargy, and anorexia (decreased suckling).

From the perspective of kinases that play integral roles in the infectious process, an extensive body of literature is available due to their critical relevance to human and animal health and their impact on gastrointestinal health. Only a salient subset of kinases are included in this article. Detailed reviews of the engagement of host kinases with RVs are available [60,61,62,63,64,65]. Infection with RVA was shown to activate receptor-interacting protein kinase 1 (RIPK1), RIPK3, and mixed-lineage kinase domain-like protein (MLKL), in infected cells, contributing to the necroptosis of the cell [65]. The NF-κB signaling cascade was also associated with RVA infection, the activation of which caused the activation of the Nod-like receptor (NLR) inflammasome response, the activation of caspase 1, and pyroptosis in intestinal cells [66]. However, there is also evidence that suggests that RVA may suppress NF-κB activation through non-structural protein 1 (NSP1), which may function like an E3 ligase and degrade the innate immune molecules associated with IFN activation. The additional RVA-mediated inhibition of the NF-κB pathway could also involve the sequestering of signaling pathway components in viroplasms [60]. Bovine RV was shown to activate calcium-/calmodulin-dependent kinase II based on the expression of the viral NSP4 protein [60]. Casein kinase II was also shown to be an essential kinase for RVA, due to its ability to phosphorylate NSP5 [67]. 

## 7. Hendra Virus (HeV)

Hendra virus (HeV) is a rare but deadly infection that causes fatal diseases in equines [68]. HeV belongs to the *Paramyxoviridae* family and emerged in 1994 as a zoonotic disease, typically spilling over from bats of the genus *Pteropus*, although other bat species have been implicated as well [68,69]. HeV was originally isolated from the infected lung tissue of a horse, during the initial outbreak that ultimately led to 14 of 21 horses dying or being euthanized [69]. Despite the limited number of cases of HeV in horses, there is mounting evidence that it can be transmitted from horses to humans. Several cases have been reported, primarily in Queensland, Australia, where close contact with infected horses has led to illness and death of handlers [70]. During the initial outbreak, two individuals fell ill with a flu-like disease, resulting in the death of one of them. HeV was identified in the kidney tissue of the fatal case [69]. Since then, 14 additional outbreaks have occurred in Australia, 5 of which have been associated with the spillover of the disease in humans [69]. HeV is believed to be transmitted to equines via flying-fox secretions, although during outbreaks, it is believed that horse-to-horse and horse-to-human transmissions are also possible. The disease manifests in equines as fever, depression, tachycardia, shortness of breath, and ataxia. The death of infected equines usually occurs in 75% of cases and within 48–72 h of symptom onset. Serological studies of equines living in Queensland have not identified them as natural reservoirs [71]. From the perspective of kinases and activated signaling responses, the data for HeV are sparse. Infection is known to modulate IFN-I responses potentially by involving the RIG-I-like receptor (RLR) pathway. Based on similarities between HeV and Nipah virus, the M (matrix) protein interacts with TRIM6, causing its degradation. TIM6 is known to catalyze the downstream activation of IKKe, thus potentially contributing to decreased IFN-I responses [72,73]. 

## 8. Equine Influenza Virus (EIV)

Equine influenza virus (EIV) is a highly contagious virus belonging to the *Orthomyxoviridae* family with a segmented negative RNA genome. Equine infection through EIV is characterized by elevated body temperature and dry cough, with severely infected horses presenting with inspiratory and expiratory wheezing and crepitations. Anorexia, lethargy, and nasal discharge are also characteristic features of EIV infection. In the context of vaccinated animals, animals usually recover within 7–10 days, while morbidity can reach 100% in non-immunized populations [74,75]. Consistent with the respiratory route of infection, the inflammation of the airways is observed, characterized by peribronchial and peribronchiolar hyperemia, mononuclear cell infiltration, the exudation of neutrophils and macrophages, and alveolar edema. In some cases, there have also been reports of myocarditis [74,76,77]. 

Although the majority of documented cases of EIV are limited to equids, there has been evidence of rare spillover incidents of EIV into other species such as dogs, pigs, and foxhounds [78,79,80,81]. It has also been demonstrated that cats experimentally infected with EIV exhibit respiratory signs with the potential of transmission to other cats [78,82]. Although the natural infection of humans with EIV has not been completely confirmed, it is believed that humans are potential hosts for EIV. In the 1960s, antibody-negative human volunteers experimentally infected with EIV demonstrated seroconversion, positive virus throat swab cultures, and shedding during days 2–6 of infection [78,83,84]. Furthermore, recent advancements in serological assays have demonstrated that individuals exposed to horses within 10 years in Iowa were more likely to have elevated antibodies against EIV, demonstrating the potential for EIV transmission to humans from horses [78,85].

Currently, the available information about kinase activation in EIV-infected host cells is limited. RIPK1/3 and MLKL proteins have been shown to be associated with necroapoptosis in the context of EIV infection [74,86]. Stress-activated kinases, including JNK/SAPK, were reported in EIV-infected MDCK cells [87]. Increased oxidative stress following EIV infection may underlie the activation of both of the above-mentioned kinases [87,88]. The oxidative stress and the associated increase in reactive oxygen species (ROS) have also been shown to be associated with c-Jun/AP-1 activation in cell culture [87]. 

## 9. Equine Herpes Virus (EHV)

EHV was first isolated as being associated with causing equine abortions [89,90]. Currently, nine EHVs are known, belonging to either the *Alphaherpesvirinae* (EHV-1, 3, 4, 6, 8, and 9) or *Gammaherpesvirinae* (EHV-2, 5, and 7) subfamilies [89,91,92]. Among the EHVs, only EHV1-5 are known to cause disease in horses, with all except for EHV-3 being associated with respiratory disease [91,93]. In alignment with being a herpes virus, latency is an important aspect of infection and epidemiology. It is believed that between 80% and 90% of infections with EHV-1 or EHV-2 usually occur before equines reach 2 years of age [89,91]. Virus reactivation from latency can occur, which will present as a clinical disease, with viral shedding [89]. EHV-1 infection is highly contagious and is acquired through contact with infectious material, including fomites and aerosols [91,94]. Horse-to-horse transmission can occur due to close contact with an infected animal or due to shedding during reactivation from latency [89]. Infected mare and foal populations serve as reservoirs. EHV-1 infects the pregnant uterus of broodmares, resulting in multifocal vasculitis, with the ultimate result being the detachment of the fetus due to the coagulation and necrosis of blood vessels. This can lead to the late-stage, spontaneous abortions of a fetus before viral infection can even be detected [80]. A clinical sequel of the respiratory version of EHV-1 is neurological disease possibly because of tropism towards endothelial cells, more than a direct neurotropic character [89,95,96,97,98]. The neurological form of the disease includes the development of vasculitis with or without hemorrhage, and thromboischemic necrosis in the brain microvasculature [89,99].

EHVs have been shown to activate several cellular kinases and signaling pathways, one of which is the NF-κB pathway. Specifically, the vCLAP protein was involved in activating NF-κB in an IKK-gamma-dependent pathway [100]. In experiments involving Chinese hamster ovary (CHO) cells, EHV-1 infection resulted in the activation of the serine/threonine Rho kinase (ROCK1), and the inhibition of this kinase by small molecules or via the overexpression of a negative regulator of ROCK1 blocked EHV-1 [101]. In conjunction with Rho kinase signaling, p38 MAPK activation was also noted in infected cells [101]. The activation of FAK and pyruvate kinase 2 (Pyk2) was shown to be required for the transport of the viral capsid protein to the nucleus [101,102]. 

Many non-equine herpesviruses and other DNA viruses (e.g., vaccinia virus) encode for viral thymidine kinases (TKs), which play important roles in the viral life cycle, specifically of significance in neuropathogenicity [103,104,105,106]. The alphaherpesvirus EHV-1 encodes for viral TK, which has been shown to increase the pathogenicity of infected mice and foals when compared to TK-deficient EHV-1 mutants [107,108].

Although the cases of EHV in humans have not been fully documented, it has been demonstrated that the potential for a crossover of species exists. In 2007, EHV-9 infection was documented in a 12-year-old polar bear with progressive encephalitis in a zoological garden located in San Diego, California [109]. It is believed that the polar bear was infected by EHV-9 from possible fomite transmission from a neighboring herd of Gravy’s zebras, demonstrating that EHV may potentially cross into other non-equid species [109]. While EHVs are not associated with human disease, their underlying mechanisms of infection become of paramount concern due to the severity of symptoms in equine populations. The latent nature of these viruses, followed by exposure to stress, can result in symptom onset or the shedding of the virus, potentially causing exposure to other equines. 

## 10. Rabies Virus

Rabies virus (RABV) is an enveloped, negative-sense RNA virus belonging to the *Rhabdovirus* family [1,2,3,4,5,6,110,111,112,113,114,115]. Infection is most often the result of a bite from a rabid animal, the site of which the virus will replicate in muscle and subepithelial cells until it reaches a high enough concentration to infect the nervous system [1,110]. RABV will then spread up through the spinal cord, where the maturation of virions occurs in the cytoplasmic space of the infected cell and little cell lysis occurs, resulting in minimal detection by the immune system. Rabies is an uncommon disease in equines with only sporadic occurrences; fatality is almost inevitable once symptoms develop. Infections are typically the result of epizootic cycles in surrounding wildlife, with transmission occurring from dog, fox, raccoon, skunk, or bat bites. An effective vaccine is widely disseminated for use in equines, constituting an inactivated virus. The disease most frequently manifests as ascending paralysis, ataxia, fever, loss of muscle tone, and death due to cardiorespiratory failure [1,110]. Rabies is a Category C zoonotic pathogen and presents a more insidious disease course in equines as compared with other mammals [5,114].

AP-2-associated kinase 1 (AAK1), a serine–threonine kinase, has been shown to mediate rabies viral entry via the phosphorylation of AP2M1 [4,113]. While the receptor-mediating entry in an AP2-dependent manner is not known, this does indicate that RABV may be able to enter host cells in a non-clathrin-dependent manner. The rabies phosphoprotein P (RABV P) is phosphorylated by host protein kinase C (PKC) isomers or heparin-sensitive kinases, with PKCγ shown to be the most effective [2,111]. Additionally, RABV P interacts with focal adhesion kinase (FAK), a protein tyrosine kinase, and the depletion of FAK in infected cells resulted in the inhibition of viral replication [3,112]. FAK plays a pivotal role in the transmission of signals between the extracellular matrix and the cytoplasm, and when activated, recruits Src kinases, which in turn fully activates FAK. This leads to the activation of further kinases downstream, such as phosphoinositol 3-kinase (PI3-K/Akt) [3,112]. RABV P also antagonizes IFN-β induction by ensuring that TANK-binding kinase 1 is not able to phosphorylate IRF-3, resulting in host cells being unable to mount a full immune response [6,7,115,116].

## 11. Borna Disease Virus (BDV)

Borna disease virus (BDV) is a non-segmented negative-strand RNA virus that is the etiological agent of Borna disease (BD), belonging to the family *Bornaviridae* within the order *Mononegavirales* [117,118]. BDV was originally perplexing to scientists due to the nuclear localization of replication and transcription. It has since been maintained as a model for the persistent non-cytolytic infection of the CNS [117,119]. BDV was originally described in the 19th century in horses and has hence been described in multiple different organisms such as sheep, cattle, cats, and ostriches [117,118,120,121,122]. Although BDV is primarily found in European countries such as Germany, Switzerland, Austria, and Britain, BDV virus-specific antibodies have been found in horses in countries around the world (e.g., Israel, Japan, China, Iran, and the United States) [123,124,125,126,127]. BDV is believed to naturally spread primarily through bodily secretions, such as nasal discharge in natural infections, and has been shown to experimentally infect animal models through multiple routes such as intranasal, intracranial, and peritoneal, ultimately reaching the brain [128,129]. Most studies investigating the pathogenesis of BDV are derived from Lewis rats and mice models, which have demonstrated that the virus most likely enters through the intranasal infection of olfactory nerve endings or potentially the trigeminal nerve [123,130,131,132,133]. Natural BDV infection can lead to death 1–4 weeks after the initial onset of symptoms in >80% of animals and typically presents with encephalomyelitis [123,134,135]. Symptoms typically vary depending on the species and age of the infected animal. In horses, however, the typical incubation period ranges from 1 to 3 months and is characterized by a progressive course of encephalitis that leads to apathy, depression, stupor, fever, anorexia, and ataxia [136,137]. Disease progression will lead to behavioral changes such as eating arrest during chewing, the leaning of the head, and the crossing of the legs to find support, as well as significant autonomic nervous system impairment [123,134,136,137]. In the final stages of BD, infected horses can experience blindness and paralysis, develop neurogenic torticollis, and ultimately die [123,137,138,139]. In some cases, persistent recurring CNS infection occurs without death or symptoms, leading to carriers of BDV [134,137].

In regard to the effects of BDV on human health, there has been much debate on the topic for decades. Due to the detection of the neurological effects of BDV infection on behavior and psyche in animals, many scientists suspected this to occur in humans as well. Therefore, early studies focused primarily on psychiatric patient cohorts, which led to debatable results. However, in 2015, a close relative of Borna disease virus 1 (BoDV-1), variegated squirrel bornavirus 1 (VSBV-1), was discovered [140,141]. Four individuals working closely with squirrels succumbed to fatal encephalitis that was suspected to be due to VSBV-1 [140,141,142]. In 2018, 3 years after the discovery of VSBV-1, the first human BoDV-1 infection was detected by two independent groups in Germany [141,143,144,145]. It was determined that five individuals were infected with BoDV-1 and that three of them received an organ donation from one infected individual. Of these five individuals, three had fatal outcomes resembling that of the dead-end hosts of natural BoDV-1 infection in animals, characterized by viral antigens and viral RNA within the brains of the deceased individuals as well as BV-reactive antibodies within cerebrospinal fluids and sera [141,142,143,144,145]. Furthermore, whole-genome sequencing identified that the virus was closely related to BDV isolates found in sheep, shrews, and horses within the same area where the donor was located (Bavaria, Southern Germany) [141,142,143,144,145]. Thus, BDV clearly still represents an important zoonotic agent for which research needs to significantly expand in the near future.

Because BDV-induced behavioral impairments are hallmark clinical manifestations of BD, many studies have aimed at studying the effects of BDV infection on neurons. It has been shown that when considering synaptic vesicle (SV) recycling as a measure of synaptic activity, BDV specifically blocks the enhancement of SV recycling and causes defects in long-term potentiation [146]. This is believed to occur due to a decrease in the phosphorylation of important regulators of SV recycling (e.g., myristoylated alanine-rich C kinase substrate (MARCKS) and Munc18-1/nSec1) by protein kinase C (PKC) via BDV interference downstream of PKC activation [146]. Further evidence suggests that this PKC-dependent phosphorylation interference may be carried out by the BDV phosphoprotein [146]. Further studies have hypothesized that persistent BDV infection leads to the interference of the response of neurons to important growth factors (e.g., proliferation, differentiation, and survival). In the neural crest-derived cell line PC12, it has been shown that PC12 cells persistently infected with BDV exhibited high levels of constitutively activated MEK1/2 and ERK1/2 in the absence of nerve growth factor (NGF) [118]. Despite the high activation levels of MEK1/2 and ERK1/2, it was noted that persistently infected PC12 cells exhibited impaired nuclear translocation of phosphorylated ERK [118]. Although the mechanism of impairment is not well understood, because BDV uniquely replicates in the nucleus, it is conceivable that BDV potentially hijacks important host factors related to nuclear translocation for its own benefit [118]. Similarly, it is well known that most of the members of the order *Mononegavirales* have phosphoproteins that play important roles as transcription factors. In the case of BDV, the phosphoprotein P is believed to be phosphorylated by protein kinase C (PKC) and casein kinase II [147,148]. In the same study, through the purification of PKC from rat brain extracts, it was determined that the main regulator of the phosphorylation of BDV P protein (BDV-P) is the protein kinase Cε (PKCε) [148]. Moreover, it was ultimately determined that this phosphorylation of BDV-P by PKC is required for efficient viral spread and dissemination [147]. Because BDV maintains the ability to persistently infect cells of the CNS, there must be a clear ability to evade the innate immune response. In this regard, recent studies have shown that the constitutive activation of NF-κB impairs BDV virus replication and that BDV-infected cells suppress the IKK/NF-κB signaling pathway downstream of IKK [149,150]. Further studies on this subject using multiple expectation–maximizations for motif elicitation (MEME) have shown that the nucleoprotein of BDV (BDV-N) and NF-κB1 share an ankyrin-like motif, which is believed to allow BDV-N to inhibit NF-κB1 processing by the 20S proteasome, ultimately leading to the inhibition of the IKK/NF-κB activation pathway [150].

## 12. Conclusions

Equine viruses constitute an important group of potential vector-transmitted zoonotic viral pathogens that are highly relevant to the One Health concept due to the disease burden and symptomology in animals and humans. Several factors contribute to the persistence and global distribution of these pathogens, with fairly limited attention paid to the development of robust therapeutic strategies to treat human exposures. There are vaccine-induced protection processes for equines for several diseases discussed here, and the possibility of spillover into humans continues to pose a significant challenge. When evaluating the interactions of several of the equine virus proteins with the host kinase machinery, some common trends emerge, including the role played by highly conserved innate immune phosphosignaling mechanisms, in the establishment of a productive infection. This suggests that the repurposing of FDA-approved therapeutics to treat human cases of equine virus infections is a viable option, with a greater chance of achieving broad-spectrum protection. However, much work is still needed to identify such broadly effective solutions to treat infections and ascertain any potential for the development of resistant variants. 

## Data Availability

Not applicable.
